# Controlling Electronic States of Few-walled Carbon Nanotube Yarn *via* Joule-annealing and *p*-type Doping Towards Large Thermoelectric Power Factor

**DOI:** 10.1038/s41598-020-64435-0

**Published:** 2020-04-29

**Authors:** May Thu Zar Myint, Takeshi Nishikawa, Kazuki Omoto, Hirotaka Inoue, Yoshifumi Yamashita, Aung Ko Ko Kyaw, Yasuhiko Hayashi

**Affiliations:** 1 Graduate School of Natural Science and Technology, Okayama University, Okayama, 700-8530 Japan; 2 Faculty of Advanced Materials Engineering, University of Technology (Yatanarpon Cyber City), Pyin Oo Lwin District, Mandalay Division, Mandalay, Myanmar; 3 Department of Electrical and Electronic Engineering, Southern University of Science and Technology, Shenzhen, 518055 P. R. China

**Keywords:** Materials science, Nanoscience and technology

## Abstract

Flexible, light-weight and robust thermoelectric (TE) materials have attracted much attention to convert waste heat from low-grade heat sources, such as human body, to electricity. Carbon nanotube (CNT) yarn is one of the potential TE materials owing to its narrow band-gap energy, high charge carrier mobility, and excellent mechanical property, which is conducive for flexible and wearable devices. Herein, we propose a way to improve the power factor of CNT yarns fabricated from few-walled carbon nanotubes (FWCNTs) by two-step method; Joule-annealing in the vacuum followed by doping with *p*-type dopants, 2,3,5,6-tetrafluo-7,7,8,8-tetracyanoquinodimethane (F4TCNQ). Numerical calculations and experimental results explain that Joule-annealing and doping modulate the electronic states (Fermi energy level) of FWCNTs, resulting in extremely large thermoelectric power factor of 2250 µW m^−1^ K^−2^ at a measurement temperature of 423 K. Joule-annealing removes amorphous carbon on the surface of the CNT yarn, which facilitates doping in the subsequent step, and leads to higher Seebeck coefficient due to the transformation from (semi) metallic to semiconductor behavior. Doping also significantly increases the electrical conductivity due to the effective charge transfers between CNT yarn and F4TCNQ upon the removal of amorphous carbon after Joule-annealing.

## Introduction

Nowadays, personnel electronic devices are ubiquitous in our daily lives. However, these devices heavily rely on batteries for operation while there are various forms of heat (energy) dissipated into our environment including our body heat as a waste^1,2^. Therefore, flexible thermoelectric (TE) device becomes a promising technology to power the personal electronic devices from surrounding waste heat. The performance of the TE materials is usually determined by the dimensionless figure of merit, *ZT* = *S*^2^ *σ T/κ*, where *S* is the Seebeck coefficient or thermopower (V K^−1^), *σ* is the electrical conductivity (S cm^−1^), *T* is the absolute temperature (K) and *κ* is the thermal conductivity (W m^−1^ K^−1^)^3^. Power factor, *P* = *S*^2^ *σ* can also be used alternatively to explain the thermoelectric performance of materials^4–7^^.^ Recently, TE performance has been optimized through formation of nanocomposite with conducting polymers, point defect engineering, energy filtering, controlling the density of state, and so on^8–10^. Consequently, many semiconductors that provide the best TE performance are costly to fabricate since they are based on inorganic counterparts. Due to the scarcity of raw materials, and toxicity, inorganic semiconductors have a lot of drawbacks for fabrication of ubiquitous TE generators^11,12^. Carbon nanotube (CNT) yarn is one of the potential ubiquitous TE materials because flexible, wearable, light-weight and robust TE devices can be easily fabricated from CNT yarn^9^. Moreover, the carrier type of the CNT yarn can be easily altered by simple doping, paving it for using in various wearable or flexible TE applications^1,13,14^. Several methods have been attempted to improve the TE performance of CNT yarn. MacLeod *et al*. have reported *n*-type and *p*-type single-walled carbon nanotube (SWCNT) thin films with TE power factors in the range of 700 µW m^−1^ K^−2^ at 298 K using a combination of ink chemistry, solid-state polymer removal, and charge transfer doping strategies^15^. An *et al*. demonstrated CNT webs with power factor of 3103 µW m^−1^ K^−2^, which is the highest among organic TE materials and comparable with that of inorganic material (Bi_2_Te_3_) at room temperature, by annealing and treatment with benzyl viologen as *n*-type dopant. As a counterpart for *n*-type CNT web, *p*-type TE power factor of 2252 µW m^−1^ K^−2^ was also reported using molecular dopant (F4TCNQ)^9^. Choi *et al*. have reported large TE power factor of 2387 and 2456 µW m^−1^ K^−2^ by *p*- and *n*-type doping of double-walled carbon nanotube (DWCNT) yarn with iron chloride and polyethylenimine, respectively^13^. In addition to pure CNT, Kim *et al*. reported SWCNT/poly (vinylidene fluoride) (PVDF) composite fibers with *p*- and *n*- type power factors of 378 ± 56 and 298 ± 98 µW m^−1^ K^−2^ respectively^16^. Recently, Zheng *et al*. designed three-dimensional thermoelectric textiles (TETs) with a high output power density of 51.5 mWm^−2^ and a high specific power of 171.7 µW(gK)^−1^ at ∆T = 47.5 K^17^. Despite high power factor, all these reports are based on CNT yarn composed of SWCNTs or DWCNTs^9,13,15–17^, which require precise control in fabrication process, and hence incurring high cost for mass production. Therefore, the application of CNT yarn made of few-walled CNTs (FWCNTs) in TE generators is more attractive to explore, compared to that of SWCNTs or DWCNTs, owing to low fabrication cost and availability of large quantity.

Here, we propose a way to improve the TE power factor of CNT yarn composed of individual CNTs (FWCNTs) by a facile two-step method; Joule-annealing in the vacuum condition followed by doping with *p*-type dopants F4TCNQ (2,3,5,6-tetrafluo-7,7,8,8-tetracyanoquinodimethane). Furthermore, we manifested the mechanism behind the improvement in Seebeck coefficient after Joule-annealing and the increase in electrical conductivity upon doping by the power-law model on relaxation time approximation to the single-band model and morphology change in CNT yarn. The improvement in TE power factor is due to the synergetic effect of Joule-annealing and doping. Joule-annealing removes amorphous carbon on the surface of the CNT yarn, which facilitates doping in the subsequent step, and leads to higher Seebeck coefficient due to the transformation from (semi) metallic behavior to semiconductor behavior. Doping with molecular dopant also significantly increases the electrical conductivity due to the effective charge transfer between CNT yarn and F4TCNQ upon the removal of amorphous carbon after Joule-annealing.

## Results

**Figure 1:**
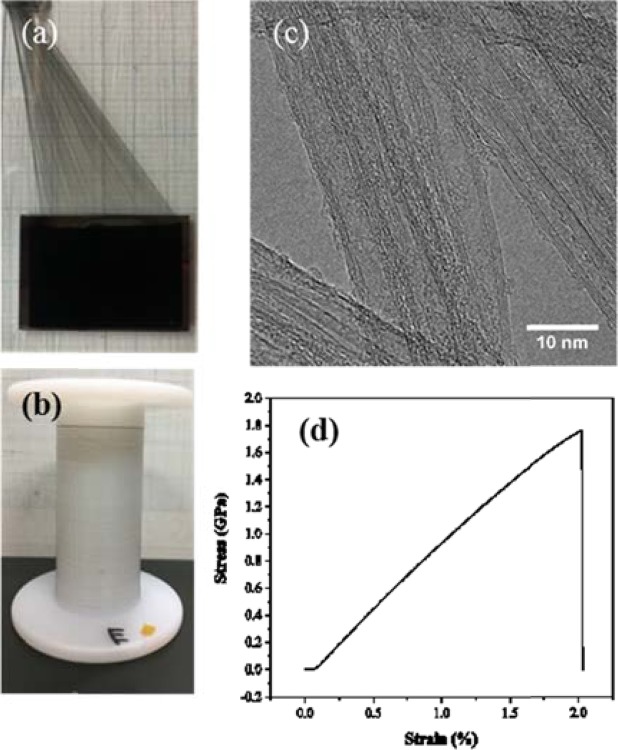
The photographs of (**a**) CNT array and web during fabrication and (**b**) prepared CNT yarn. (**c**) TEM image and (**d**) stress-strain diagram of pristine CNT yarn.

High density and vertically aligned CNT array and CNT web are shown in Fig. 1(a). The photograph of CNT yarns produced by spinning of these arrays is depicted in Fig. 1(b). CNT yarn composed of thousands of individual CNTs with tube diameter of 4‒6 nm and wall number of approximately 2‒6 was fabricated from the vertically aligned CNTs, which is observed by TEM as shown in Fig. 1(c). Pristine CNT yarn has tensile stress of 1.8 GPa at a strain of 2% (Fig. 1(d)), which is promising for flexible thermoelectric devices.

**Figure 2:**
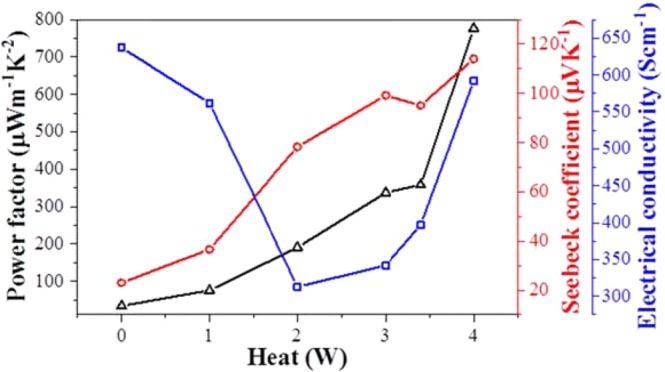
Electrical conductivity, Seebeck coefficient and power factor of CNT yarns as a function of heat amount during Joule-annealing.

The electrical conductivity, Seebeck coefficient, and power factor of pristine and Joule-annealed CNT yarns at various heating powers are shown in Fig. 2. The pristine CNT yarn has the electrical conductivity, Seebeck coefficient and power factor of 637 S cm^−1^, 23 µV K^−1^ and 34 µW m^−1^ K^−2^ respectively, at room temperature. As increasing the amount of heat, the Seebeck coefficient continuously increases up to 4 W (73 mA × 56 V). However, the electrical conductivity decreases initially until the heat amount of 2 W and increases again at the heat amount between 2 and 4 W. Consequently, after Joule-annealing at the heat amount of ~ 4 W, the Seebeck coefficient improves to 114 µV K^−1^ while the electrical conductivity becomes slightly lower than that of pristine one (592 S cm^−1^), resulting an increase in power factor from 34 to 776 µW m^−1^ K^−2^ at room temperature. It is worth mentioning that CNT yarn, however, was broken when continuously heating to 5 W.

**Figure 3:**
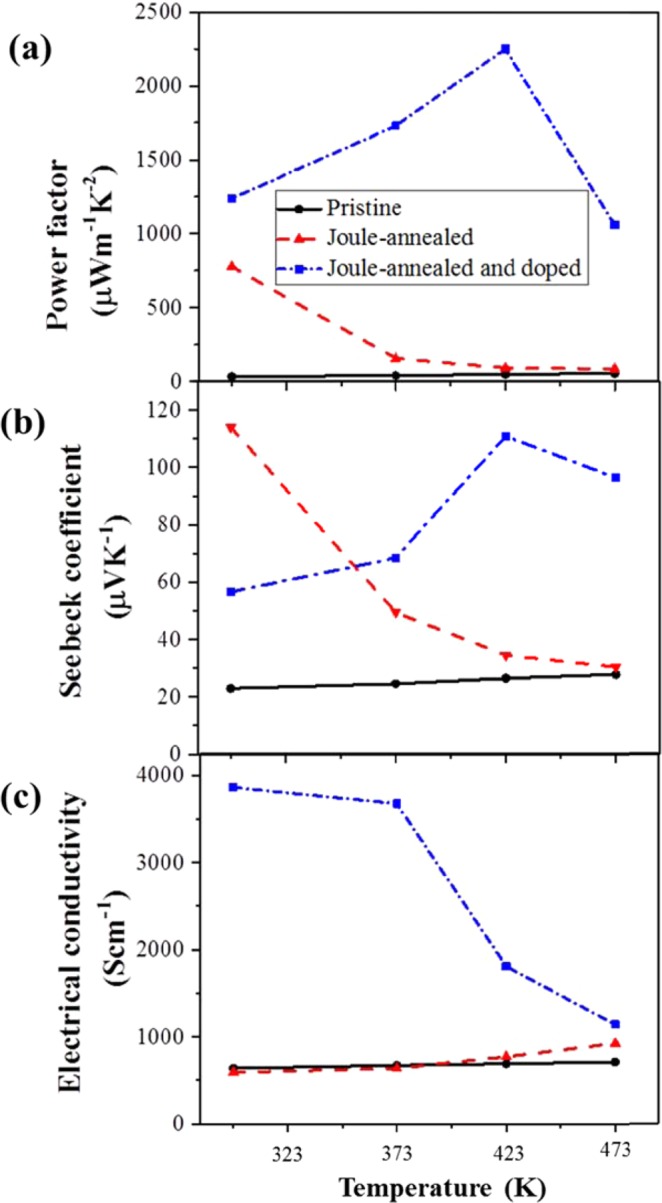
(**a**) Power factor, **(b)** Seebeck coefficient and **(c)** electrical conductivity of pristine, Joule-annealed, and Joule-annealed followed by F4TCNQ-doped (25 mg/ml) CNT yarns as a function of measurement temperatures.

**Figure 4:**
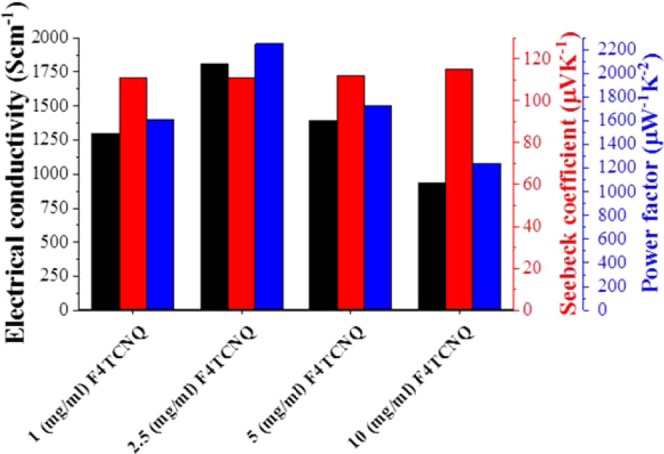
Electrical conductivity (black column), Seebeck coefficient (red column) and the corresponding power factor (blue column) of Joule-annealed CNT yarns doped with various concentrations of F4TCNQ. All samples were measured at 423 K.

Although Joule-annealing improves the Seebeck coefficient significantly, its effect is very little on the electrical conductivity. Therefore, the Joule-annealed CNT yarns are further doped with *p*-type dopants (F4TCNQ) to improve their electrical conductivity. As shown in Fig. 3, the temperature-dependent electrical conductivity and Seebeck coefficient reveals interesting trends after Joule-annealing followed by doping. Pristine CNT yarn has electrical conductivity of 637 S cm^−1^ at room temperature and it barely changes to 709 S cm^−1^ at 473 K while its Seebeck coefficient is 23 µV K^−1^ at room temperature and increases to 28 µV K^−1^ at 473 K. Therefore, the pristine CNT yarn shows (semi) metallic behavior^18^. However, the electrical conductivity increases whereas Seebeck coefficient decreases with increasing temperature after Joule-annealing^19^. Therefore, it can be attributed that CNT yarn transforms from metallic to semiconducting behavior^20^ after Joule-annealing. Moreover, generally semiconducting CNT yarn shows a larger magnitude of Seebeck coefficient than metallic CNT yarn at room temperature^21^, further supporting the argument that semiconducting behavior is dominated in CNT yarns upon Joule-annealing. In contrast, when the Joule-annealed CNT yarn is further doped with 2.5 mg/ml F4TCNQ, the electrical conductivity decreases with increasing temperature (3861 S cm^−1^ at room temperature *vs* 1142 S cm^−1^ at 473 K), whereas the Seebeck coefficient increases with increasing measurement temperature (57 Scm^−1^ at room temperature *vs* 96 Scm^−1^ at 473 K), implying that the Joule-annealed followed by doped CNT yarns return to (semi) metallic behavior as in pristine samples. The optimum power factor is obtained at a measured temperature of 423 K. Furthermore, the Joule-annealed CNT yarn is doped with various concentrations of F4TCNQ such as 1, 2.5, 5 and 10 mg/ml to investigate extensively the doping effect on the thermoelectric properties of *p*-doped CNT yarn. The optimum condition is obtained by doping with 2.5 mg/ml F4TCNQ and the electrical conductivity decreased again at higher concentrations of F4TCNQ. This is probably due to the aggregation of F4TCNQ on the surface of the CNT yarn, which is observed by SEM as in Figure S1. The electrical conductivity, Seebeck coefficient and power factor of Joule-annealed CNT yarns doped with various concentrations of F4TCNQ are illustrated in Fig. 4. All measurements were done at 423 K (optimum measurement temperature). When the CNT yarns are straight away doped with 2.5 mg/ml F4TCNQ without prior Joule-annealing, neither significant improvement in electrical conductivity (746 S cm^−1^) nor Seebeck coefficient (28 µV K^−1^) is observed, resulting in the power factor of only 60 µW m^−1^ K^−2^ at a measurement temperature of 423 K.

This suggests that Joule-annealing can facilitate doping by removing amorphous carbon on the surface of the CNT yarn, which will be discussed in the following section.

## Discussion

**Figure 5:**
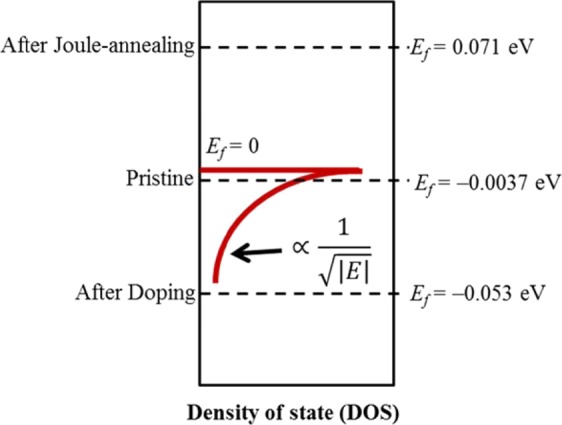
Model of single band diagram of FWCNT yarn based on calculated *E*_*f*_ values.

**Figure 6:**
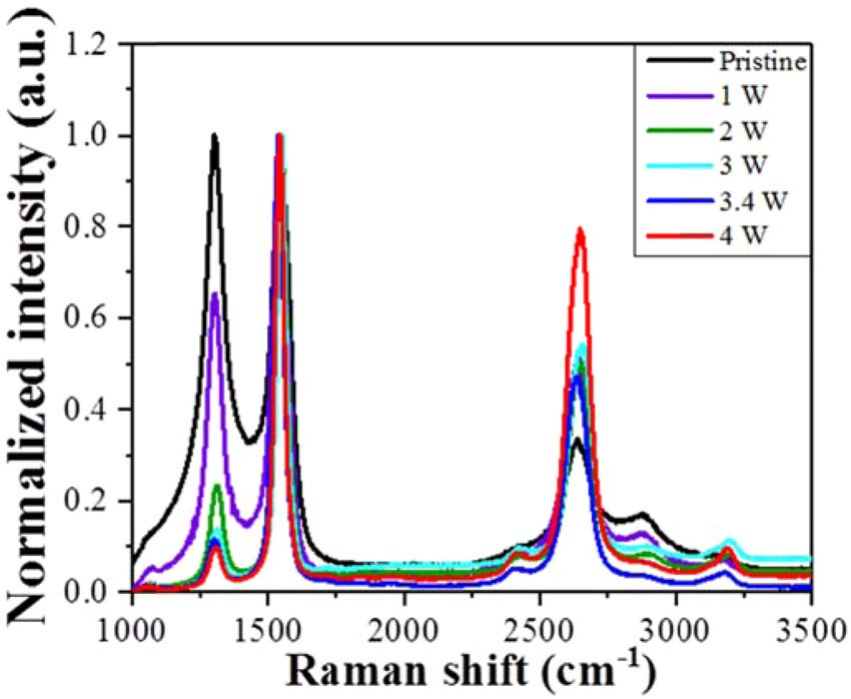
Raman spectra of pristine and Joule-annealed CNT yarns at various amounts of heat.

The significant improvement of the Seebeck coefficient and electrical conductivity after Joule-annealing followed by *p*-type doping can be qualitatively explained by applying the power-law model on relaxation time approximation to the single band. Using this model, the Seebeck coefficient of *p*-type carrier can be express as

1 $$S=\frac{{k}_{{\rm{B}}}}{e}\left[\left(\frac{d}{2}+r+1\right)-\frac{{E}_{f}}{{k}_{{\rm{B}}}T}\right]$$

where, *S* is the temperature dependent Seebeck coefficient, *k*_B_ is the Boltzmann constant*, e* is the elementary charge, *d* is the dimension of the material (1, 2, 3), *r* is a characteristic exponent of the relaxation time, *T* is the temperature at which Seebeck coefficient is measured and *E*_*f*_ is the Fermif energy, respectively. In the model, potential energy, *E* is defined by that of electron, and *E* = 0 is set to be valence band top^22^. According to Equation 1 we obtained the values of Fermi energy (*E*_*f*_) and scattering parameter (*r*) when we choose the dimension of material to *d =* 1 (one-dimensional density of state)^23^, as shown in Fig. 5. Fitted values of temperature dependent Seebeck coefficient used for this calculation are shown in Figure S2(a),(b) and (c). To better understand the numerical results, the calculated Fermi energy values are shown on the parabolic single-band as in Fig. 5. Fermi energy of the pristine CNT yarn is −0.0037 eV, which is very near to the top of the valence band. This implies that there are acceptors in the pristine CNTs which may be considered as defects introduced during the manufacturing process. After Joule- annealing, Fermi level raises up to 0.071 eV and it behaves like intrinsic semiconductor, dominating phonon scattering. Therefore, Seebeck coefficient improves significantly after Joule-annealing. After doping, the location of the Fermi level moves down to −0.053 eV. This means that electrons are caught from the CNT yarn by the dopant F4TCNQ, thus graphene-like surface of the CNT yarn is ionized and scattering process of carrier is changed from phonon to ion that increases the electrical conductivity again. Raman spectra which suggests the existence of graphene-like layer on the surface of the CNT yarn is shown in Fig. 6. Therefore, significant improvement of Seebeck coefficient after Joule-annealing and the higher electrical conductivity after doping can be explained qualitatively from Equation 1 and the single band diagram and further supported by measured temperature-dependent Seebeck coefficient. On the other hand, the calculated scattering parameter, *r* has negative value of −1.1 for pristine and it also has negative value of – 3 after Joule-annealing. This value changed to positive value of 1.14 after doping. In ideal, scattering parameter have values from *r* = −1/2 for the acoustic phonon scattering to *r* = 3/2 for ionized impurities scattering^24,25^. There is relatively large deviation for the scattering parameter values of pristine and Joule-annealed CNT yarn in this system. Even it is not enough for discussing the values of scattering parameter by this simple model, we can conclude that ionic scattering process is reduced by Joule-annealing and is enhanced by F4TCNQ doping.

To further understand the morphology-change in CNT yarns and associated change in thermoelectric properties, we also investigated Raman spectra of pristine and Joule-annealed samples. Raman spectra were scanned to capture G band (1545 cm^−1^), D band (1303 cm^−1^) and 2D band (2641 cm^−1^). G band represents in-plane bond stretching vibrations of sp^2^ hybridized carbon atoms of *E*_*2g*_ symmetry. D band comes from disorder or impurities in the CNTs such as amorphous carbon with sp^3^ bonding and 2D band is the second order vibration mode of D band and related to structural defects in continuous graphitic sheet of the nanotubes^9,19,26^. Raman spectra of CNT yarns annealed at various amount of heat are indicated in Fig. 6. It can be seen that the intensity ratio of I_G_ (G band) to I_D_ (D band) increases from average values of 1 for the pristine CNT yarns to 11.2 and 17.8 for Joule-annealed samples at 3 W and 4 W, respectively (Table S1 and Figure S3). But no Raman peak shift of the D band was observed after Joule-annealing which is consistent with other reports^19^. These results reveal the removal of amorphous carbon and impurities as well as improvement of crystallinity^27^. Concurrently, the intensities ratio of I_2D_ (2D band) to I_G_ in Raman spectra gradually increases while the amount of heat increases (Figure S3). DiLeo *et al*. have reported that 2D band is the indication of long-range order and the intensity ratio of I_2D_ to I_G_ is a more accurate measurement of CNT quality and more sensitive than the intensity ratio of I_G_ to I_D_^28^. After Joule-annealing at 4 W, the intensity ratio of I_2D_ to I_G_ reaches nearly 0.8, which points out the formation of a few layer graphene structure on the surface of CNT yarn^29^. On the other hand, since CNTs are rolled up from multi-layered graphene sheets^30^, the improvement in the intensity ratio of I_2D_ to I_G_ may indicate that Joule-annealing induces the decrease in graphite layers due to the wall-by-wall breakdown of CNTs after Joule-annealing^31^, but which is still not clear. Based on the Raman spectra, we suggest the following morphology changes in CNT yarn. After Joule-annealing, some part of amorphous carbon on the surface of the CNT yarn changed to graphene-like structure and some of the defective six-membered ring carbon atoms are also cured, which are confirmed by Raman spectroscopy as in Fig. 6. Because of the formation of graphene-like structure on the surface of the CNT yarn, dopant materials can be easily attached to the surface of the CNT yarn. Nevertheless, amorphous carbon and other impurities may decrease the carrier mobility which causes scattering and contact resistance^19^. Furthermore, electrical conductivity slightly increases again beyond 2 W because of the formation of graphene-like structure and removal of amorphous carbon. Possible mechanism of morphology change after Joule-annealing and *p*-doping is illustrated in Figure S4.

**Figure 7:**
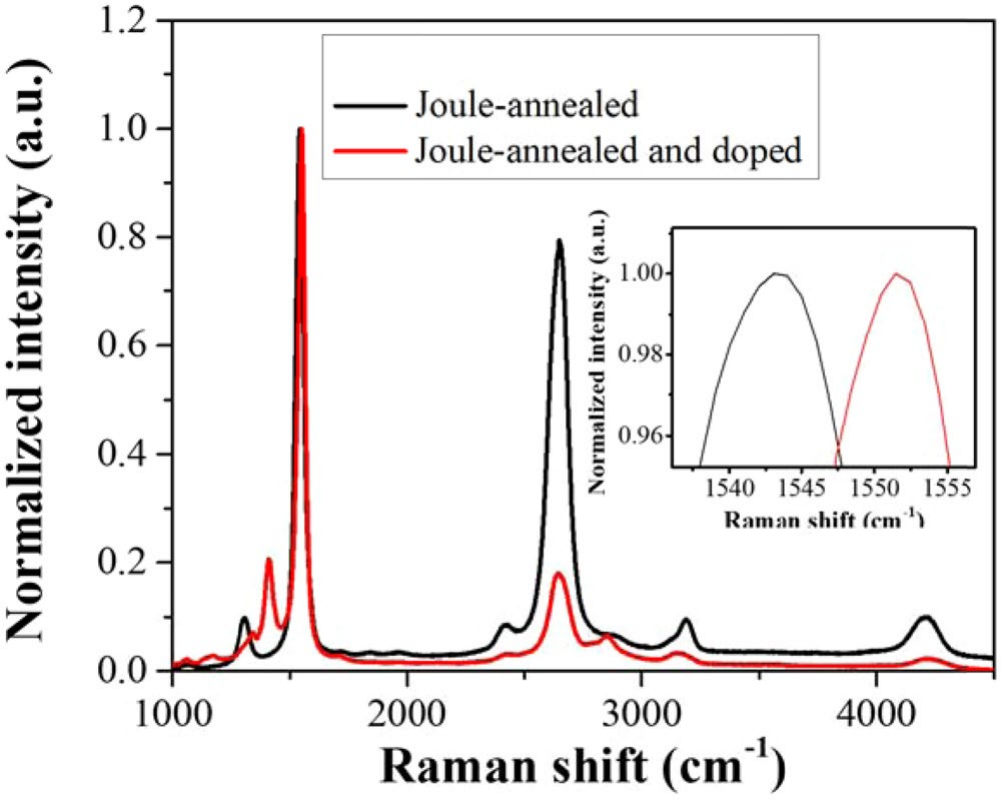
Raman spectra of Joule-annealed CNT yarn, and Joule-annealed CNT yarn doped with 2.5 mg/ml F4TCNQ. Inset is enlarged view of the Raman shift at 1540‒1555 cm^−1^.

Since the G peak of CNT corresponds to doping and upshifts for both holes and electron doping^32^, we examined the G band shift and the intensity ratio of I_G_ to I_D_ and I_2D_ to I_G_ in the Raman spectra^9^ as demonstrated in Fig. 7 to confirm the doping in CNT yarns. G band of the Joule-annealed and F4TCNQ (2.5 mg/ml)-doped CNT yarns is red-shifted^33^ from 1544 to 1552 cm^−1^. This G band shift indicates the charge transfer from CNT yarn to dopant materials with the improvement of electron-phonon coupling^26–28,32^. On the other hand, it can also be attributed to the phonon stiffening after doping. Grimm *et al*. reported that a pronounced phonon softening of the G^+^ mode is observed for low to medium hole concentrations whereas phonon stiffening is observed for larger hole concentrations^34^. Ferrari proved that doping induces a stiffening of the Raman G peak for both holes and electrons^32^. Thus, our Raman results agree well with the previous reports^32,34^. After doping, the intensity ratio of I_G_ to I_D_ and I_2D_ to I_G_ decrease again, implying that the dopants were successfully incorporated into the CNT yarn. Moreover, the new peaks appeared at around 1345 and 1414 cm^−1^ after doping may come from the F4TCNQ^9,35^. Raman spectrum of pristine F4TCNQ is acquired as in Figure S5 to explore systematically before and after doping of CNT yarn.

Morphology of pristine, Joule-annealed, and Joule-annealed followed by doped CNT yarn was observed by TEM as in Figure S6. Amorphous carbon can be seen on the surface of the pristine CNT yarn as shown in Figure S6(a). After Joule-annealing, similar morphology of the CNT yarn with the pristine one is observed as in Figure S6(b). This may be ascribed as the formation of a few-layered graphene structure on the surface of CNT yarn resulting from Joule-annealing, which agrees well with the Raman results. After Joule-annealing followed by doping, thicker coating layers observed in Figure S6(c) can be attributed to the incorporating of F4TCNQ. SEM images are also captured as in Figure S1 to get more information about morphology. After joule annealing, swelling structure is observed as demonstrated in Figure S1(b). Even though smooth surface is examined after Joule-annealing followed by doping with 2.5 mg/ml F4TCNQ (Figure S1(c)), some aggregates can be seen after doping with 10 mg/ml F4TCNQ (Figure S1(d)). This is one of the possible reasons why electrical conductivity decrease again at higher concentration of F4TCNQ.

TGA (in air) analysis was used to analyze CNT and the removal of impurity contents based on the weight changes in the CNT yarn before and after Joule-annealing. The CNT content at 450 °C is 92% for pristine and 97% after Joule-annealing, which is illustrated in Figure S7. A majority of the CNT yarn impurities such as amorphous carbonaceous impurities are lost by 450 °C in TGA^36^. This weight-loss by 450 °C can be attributed to the removal of amorphous carbon. Therefore, very less amount of amorphous carbon is present in the CNT yarn after Joule-annealing. The temperature at which CNT yarn combustion occurs (*T*_*c*_) is obtained from the TGA as 629 °C for pristine and 739 °C for Joule-annealed CNT yarn. After Joule-annealing, *T*_*c*_ upshifts with ∆Tc ≈110 °C. Large amount of *∆T*_*c*_ value indicates that CNT themselves could participate in burning process associated with Joule-annealing, leading to structure changes on CNT surfaces that effect *T*_*c*_ and hence, *∆T*_*c*_^37^.

It is worth discussing briefly the thermal conductivity of the CNT yarns although this is not within the scope of this study. Nevertheless, thermal conductivity of CNT yarn varies from the level of thermal insulation to high values depending on their structure and measurement techniques^38^. We determined the thermal conductivities of pristine and Joule-annealed CNT yarn from thermal diffusivity measured by modified AC calorimetry^39^. The thermal conductivity values are 26 and 73 Wm^−1^ K^−1^ for pristine and Joule-annealed CNT yarn, respectively. Therefore, thermal conductivity increases three times after Joule-annealing. On the other hand, CNT yarns strongly deviate from Wiedemann-Frantz law and thermal conduction mainly occurs by phonon transport in CNT yarns^40^. Hence, there are no substantial effects on phonon transport by doping which cannot alter the bonding condition^41^. Thus, thermal conductivity of Joule-annealed and doped CNT yarn is considered to be similar to that of the Joule-annealed CNT yarn. Based on this estimation, thermoelectric figure of merit of Joule-annealed followed by doped CNT yarns is about 0.013 while that of only Joule-annealed CNT yarns is 0.0005.

It has been known that the best TE materials would behave as a “*phonon-glass/electron-crystal* (PGEC)”; that means it would have the electrical properties of a crystalline material and the thermal properties of an amorphous or glass-like material^41,42^. Despite the fact that the power factor of FWCNT yarn is significantly increased by Joule-annealing followed by doping, we postulate that it is still far from “*phonon-glass/electron-crystal* (PGEC)” behavior. The thermal conductivity increases three times upon Joule-annealing, which obviously deviates from phonon glass behavior. On the other hand, doping does not induce substantial effect on phonon transport due to unchanged bonding condition despite considerable improvement in electrical conductivity, bringing closer to the PGEC behavior for doped CNT yarns. Recent theoretical and experimental study revealed that there is lack of thermal percolation in carbon nanotube composites at low nanotube loading although steep increase in electrical conductivity is observed^43,44^. Therefore, incorporating Joule-annealed and doped CNT yarns in polymer metrix (at low loading) could be an option to make PGEC.

In conclusion, a simple and effective way for *p*-type doping of FWCNT yarn with F4TCNQ is presented in this study. Numerical approach how Seebeck coefficient increased significantly after Joule-annealing and how electrical conductivity increased significantly after doping are also discussed. Our results show that doping after Joule-annealing can lead higher thermoelectric power factor of CNT yarn. Specifically, FWCNT yarn doped with 2.5 mg/ml F4TCNQ exhibited a power factor of 2250 µW m^−1^ K^−2^, which is one of the highest values among the *p*-typed doped CNT yarn. Moreover, pretreatment by Joule-annealing is confirmed to be effective way to improve Seebeck coefficient due to the transformation of (semi) metallic to semiconductor behavior. Finally, combination of Joule-annealing and doping can pave a way to enhance the thermoelectric properties of CNT yarn.

## Methods

### Experimental details

Chemical vapor deposition (CVD) method (Black Magic II, Aixtron Ltd.) was used to synthesize high-density and vertically aligned CNT array. A thin layer of iron was used as a catalyst which was deposited on the substrate (Al_2_O_3_/SiO_2_/Si) by electron beam deposition (VTR-350M/ERH, ULVAC KIKO Inc.)^45^. Then, FWCNT yarns (15‒20 µm) were fabricated from the CNT array using dry spinning method with rotation speed of 500 rpm and drawing speed of 300 mm/min. Joule-annealing was accomplished by passing a current of ~ 73 mA and voltage of ~56 V under vacuum condition at around 1‒2 × 10^−4^ Pa for 1 min^39,46^.

F4TCNQ was purchased from Luminescence Technology Corporation. Chlorobenzene (99%) was purchased from Wako Pure Chemical Industries, Ltd. F4TCNQ was dissolved in chlorobenzene solution by probe sonication for 10 min followed by magnetic stirring at 110 °C for 20 min at 300 rpm. Doping was performed with various concentrations of F4TCNQ such as 1, 2.5, 5 and 10 mg/ml respectively. Then CNT yarns were dipped into F4TCNQ solution^47,48^ for 6 hrs. As a consequence, CNT yarns were dried in an oven at 80 °C for 15 min.

### Measurement and characterization

ZEM-3M8 ULVAC (commercially available) Seebeck Coefficient Measurement System (ADVANCED RIKO, Inc) was used to measure the thermoelectric properties such as Seebeck coefficient, electrical conductivity and power factor, which has a special sample-attachment to measure the thin film or wire specimen. Measurement was performed in the helium environment. Transmission electron microscopy (TEM) (JEOL, JEM-2100F) was used to analyze the number of walls of CNTs and the presence of amorphous carbon surrounding CNTs. Tensile strength was measured with Autograph, AGS-X, 5 N (Shimadzu). Analytical scanning electron microscope (SEM, JEOL, JSM-6060LA) was used to measure the diameter of CNT yarns as well as to analyze the surface condition of the CNT yarn. Raman spectroscopy (JASCO, NRS4500 NMDS) was performed using 532 nm excitation (green) lasers to characterize short/long-range order/disorder in CNT yarns. The presence of carbonaceous entities before and after Joule-annealing was quantitatively estimated by thermal gravimetric analysis (TGA‒DTG-60, SHIMADZU). TGA was performed at 10 °C/min from 25 to 1000 °C under air flow rate of 300 ml/min. Modified alternative current (AC) calorimetry (Laser PIT, Ulvac) was used to measure the thermal diffusivity of the CNT yarn and measurement procedure are the same as in Hada *et al*.^39^.

## Supplementary information

Supplementary information.
