# Supplementary material for: Controlling Electronic States of Few-walled Carbon Nanotube Yarn via Joule-annealing and p-type Doping Towards Large Thermoelectric Power Factor
Source: Sci Rep. 2020 Apr 29;10:7307. doi: 10.1038/s41598-020-64435-0 (PMC7190723; doi:10.1038/s41598-020-64435-0)
Supplement: Supplementary file 1 — Supplementary information. [file 41598_2020_64435_MOESM1_ESM.docx]

**Supplementary Information**

Controlling Electronic States of Few-walled Carbon Nanotube Yarn via Joule-annealing and *p*-type Doping Towards Large Thermoelectric Power Factor

**May Thu Zar Myint^a,b^, Takeshi Nishikawa^a^, Kazuki Omoto^a^, Hirotaka Inoue^a^, Yoshifumi Yamashita^a^, Aung Ko Ko Kyaw^c^*,Yasuhiko Hayashi^a^***

^a^ Graduate School of Natural Science and Technology, Okayama University, Okayama, 700-8530, Japan.

^b^ Faculty of Advanced Materials Engineering, University of Technology (Yatanarpon Cyber City), Pyin Oo Lwin District, Mandalay Division, Myanmar.

^c^ Department of Electrical and Electronic Engineering, Southern University of Science and Technology, Shenzhen 518055, P. R. China.

E-mail: [hayashi.yasuhiko@okayama-u.ac.jp](mailto:hayashi.yasuhiko@okayama-u.ac.jp) and [aung@sustech.edu.cn](mailto:aung@sustech.edu.cn)

**Contents**

*Figures*

Figure S1: SEM images of pristine, Joule-annealed and Joule-annealed followed by F4TCNQ doped CNT yarn.

Figure S2: Fitted curves of the temperature dependence Seebeck coefficient for pristine, Joule-annealed and Joule-annealed followed by F4TCNQ doped CNT yarn.

Figure S3: Relationship between the amount of joule-annealing and the intensity of 2D band to G band.

Figure S4: Possible mechanism of morphology-change after Joule-annealing and doping.

Figure S5: Raman spectrum of F4TCNQ.

Figure S6: TEM images of pristine, Joule-annealed and Joule-annealed followed by
 F4TCNQ doped CNT yarn.

Figure S7: TGA analysis of the CNT yarn before and after Joule-annealing.

*Tables*

Table S1: The values of the intensity ratio of I_G_/I_D_ and I_2D_/I_G_.


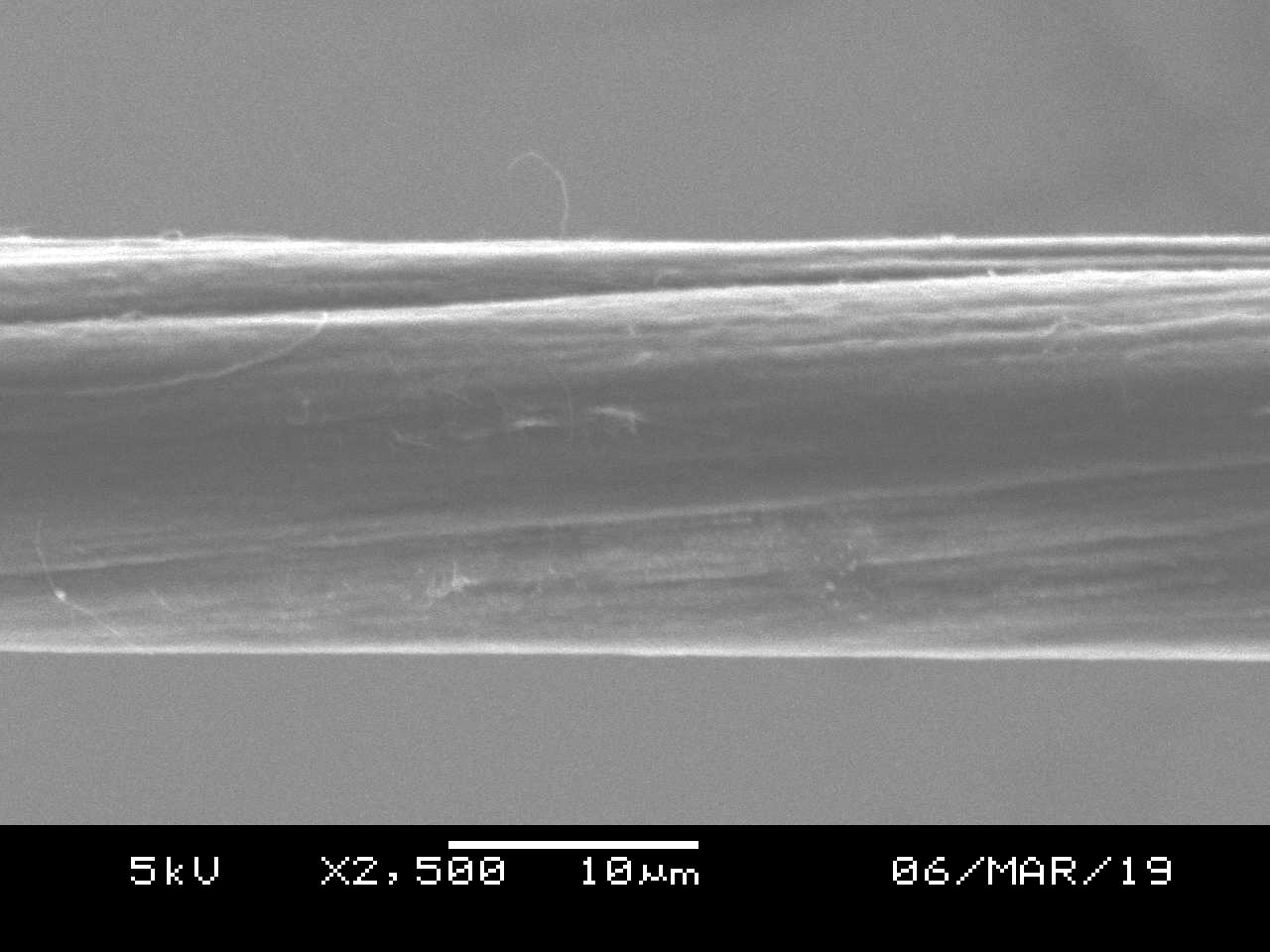


**Pristine**

**(a)**


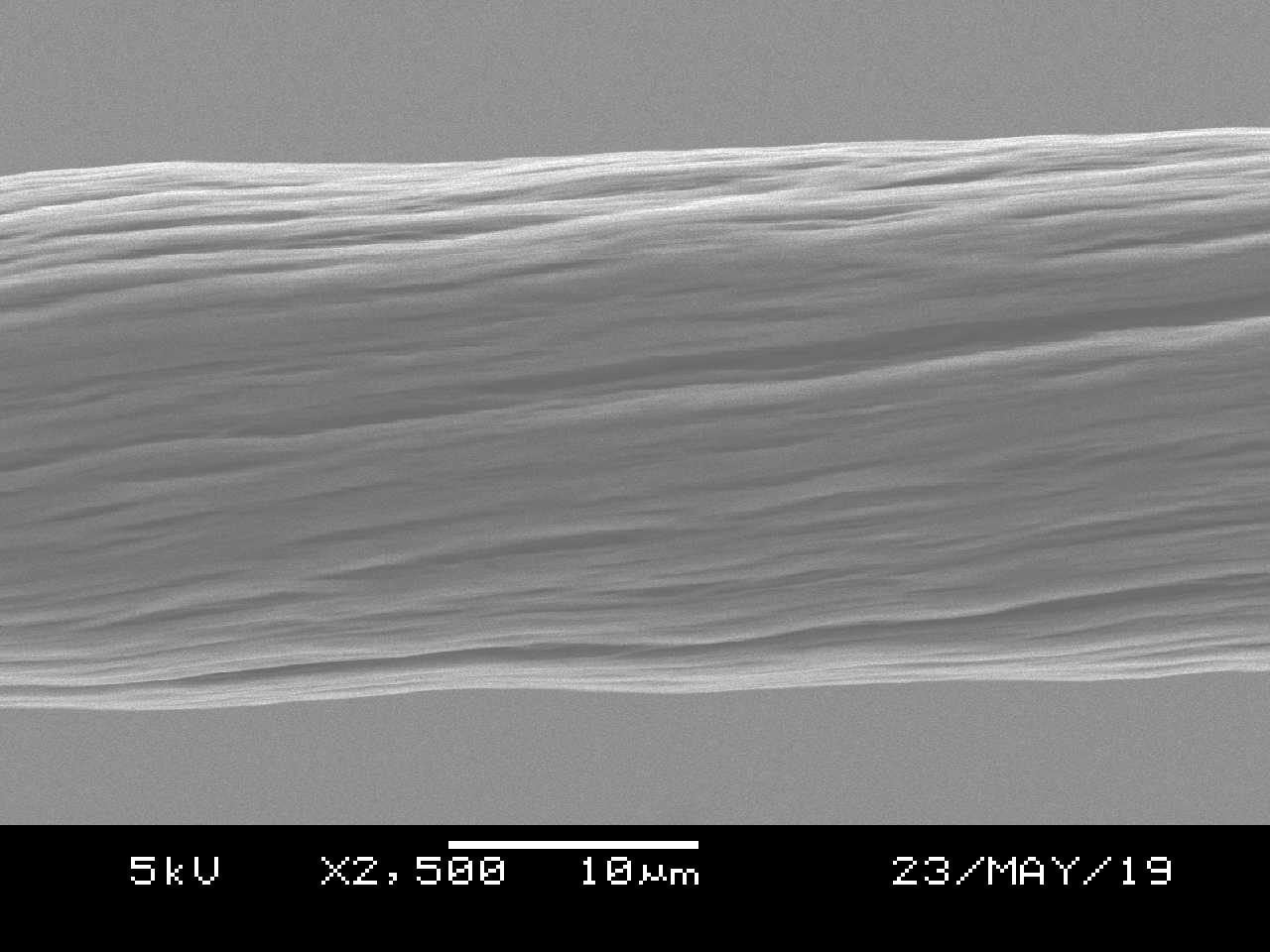


**Joule-annealing**

**(b)**


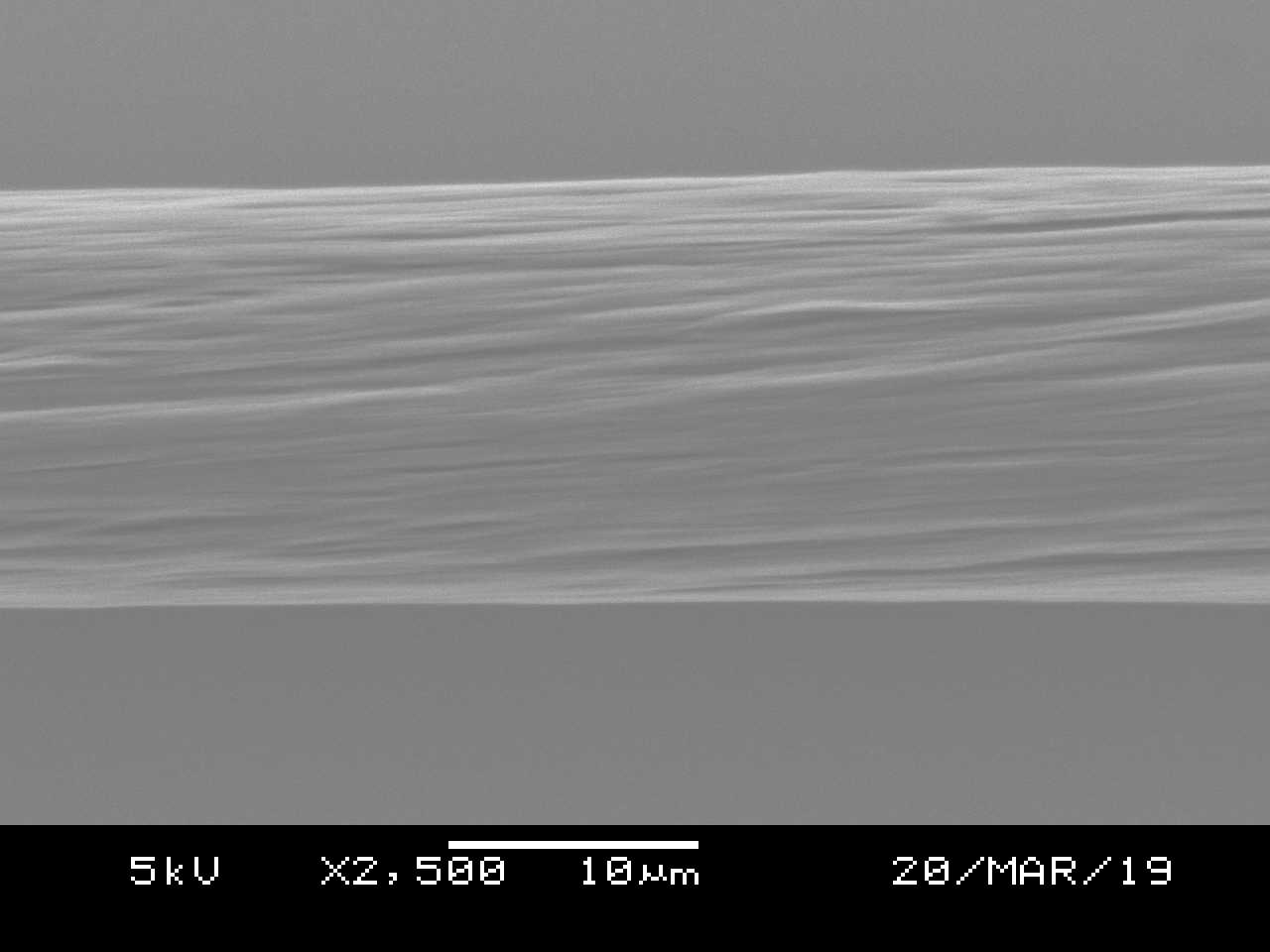


**2.5 mg/ml F4TCNQ doped**

**(c)**


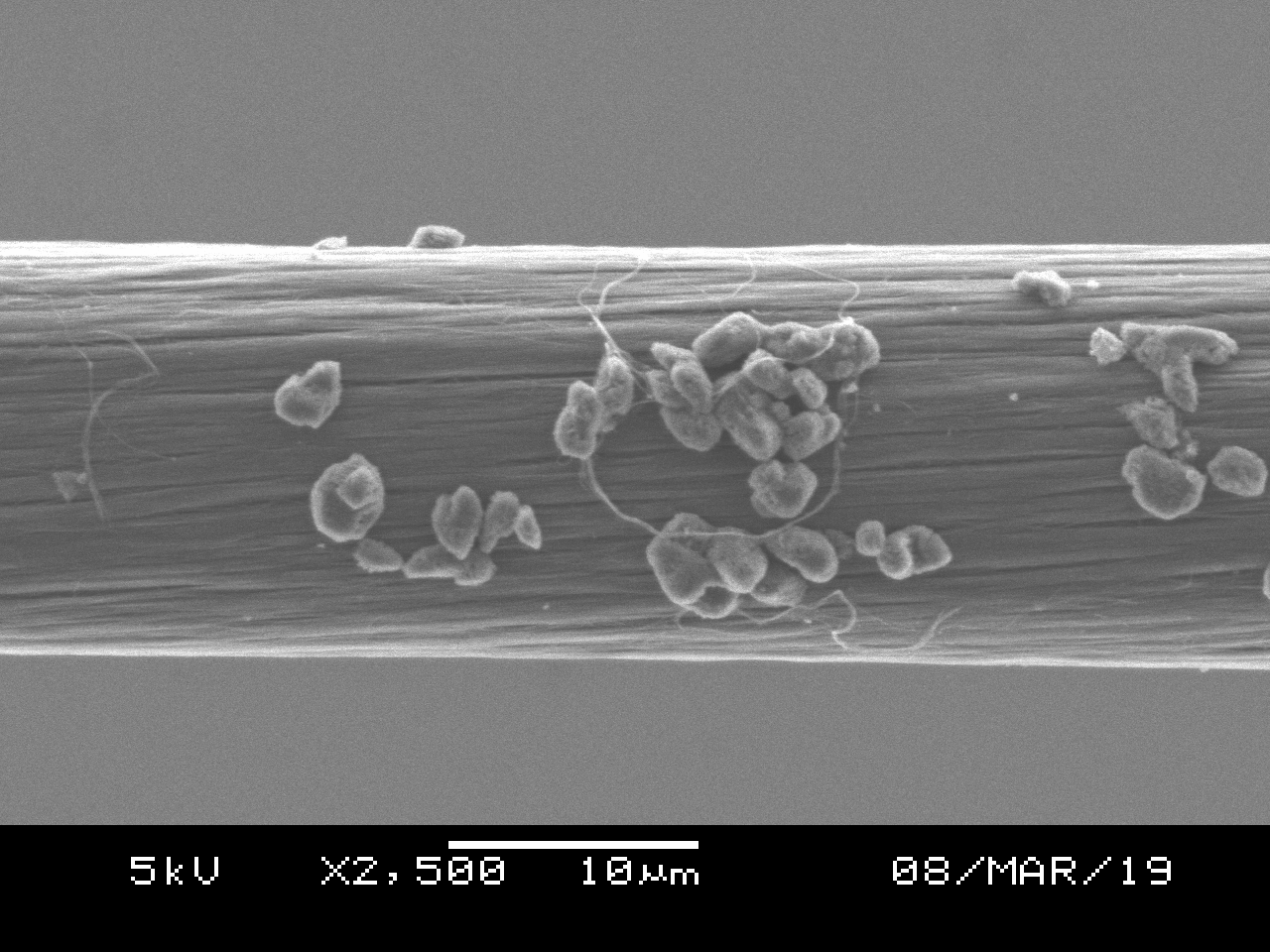


**10 mg/ml F4TCNQ doped**

**(d)**

Figure S1: SEM images of (a) pristine, (b) Joule-annealed, (c) Joule-annealed followed by 2.5 mg/ml F4TCNQ doped, and (d) Joule-annealed followed by 10 mg/ml F4TCNQ doped FWCNT yarns.

Table S1: The values of the intensity ratio of IG/ID and I2D/IG.

| Joule Heat (W) | I_G_/I_D_ | I_2D_/I_G_ |
| --- | --- | --- |
| 0 (pristine) | 1 | 0.3 |
| 1 | 1.6 | 0.5 |
| 2 | 5 | 0.5 |
| 3 | 11.2 | 0.5 |
| 3.4 | 15.7 | 0.4 |
| 4 | 17.8 | 0.7 |


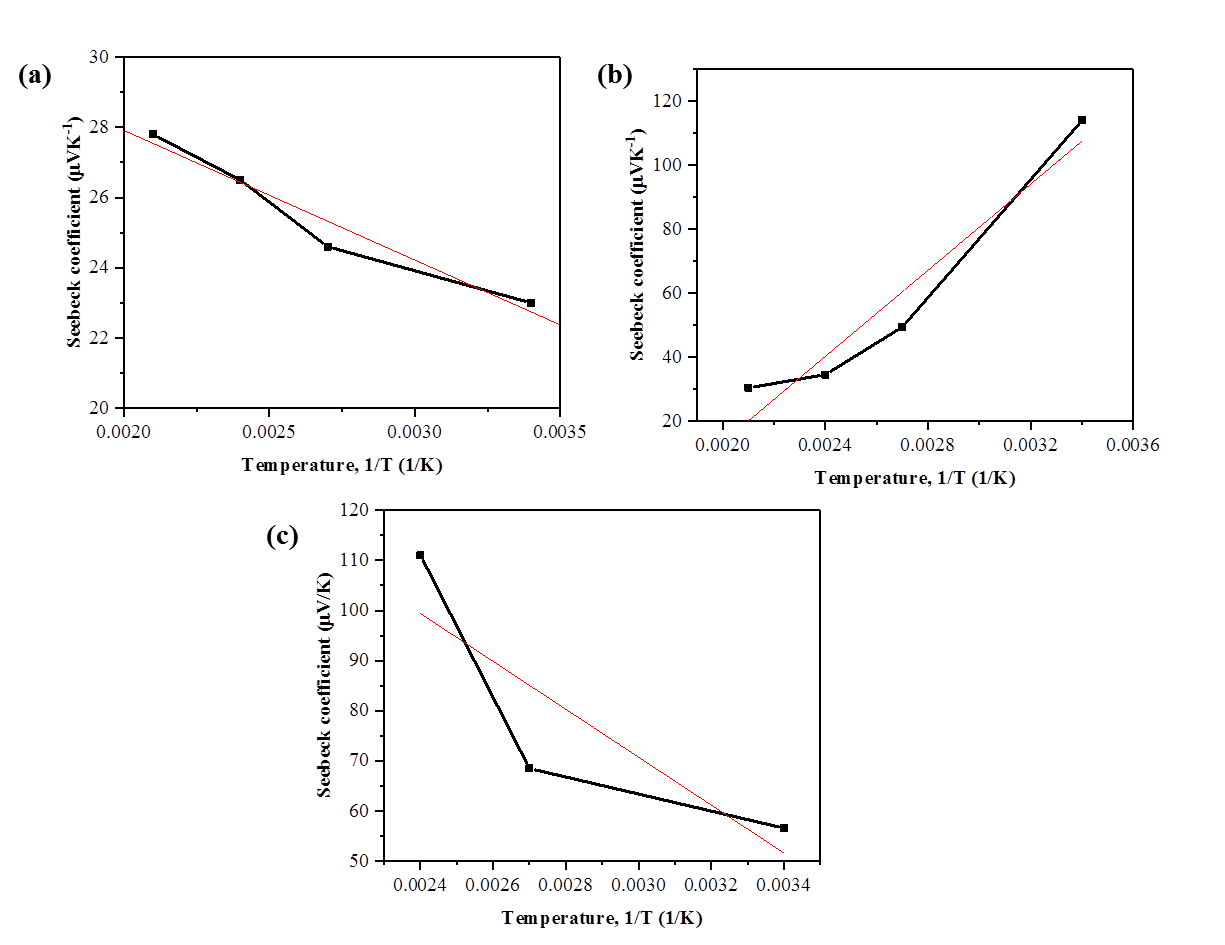


Figure S2: Fitted curves of the temperature dependent Seebeck coefficient for (a) pristine, (b) Joule-annealed and (c) Joule-annealing followed by F4TCNQ doped FWCNT yarns.


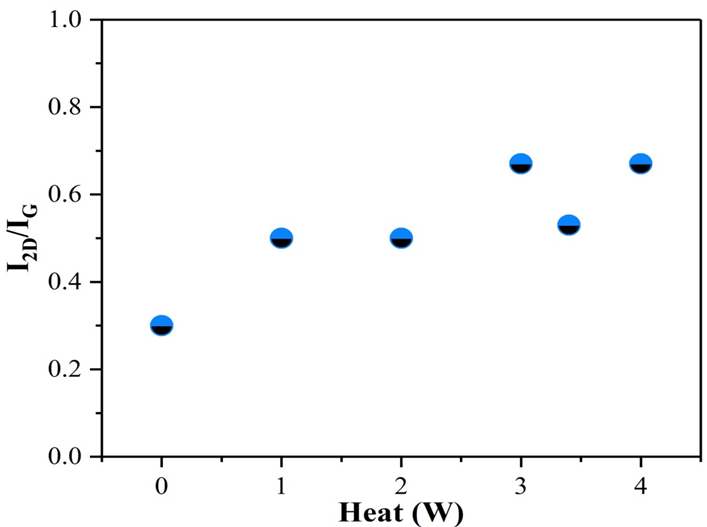


Figure S3: Relationship between the amount of joule-annealing and the intensity of 2D band to G band in Raman spectroscopy, indication of the formation of multi-layered graphene structure on the surface of FWCNT yarn.


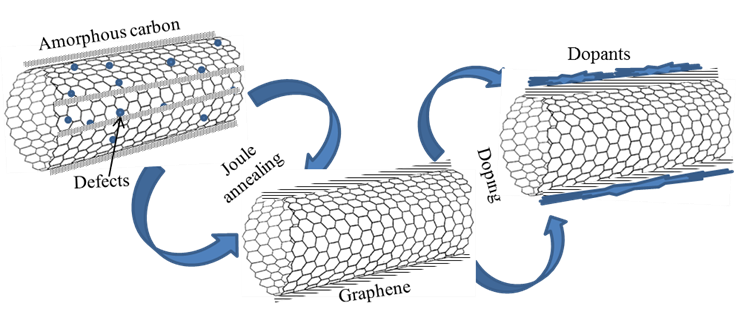


Figure S4: Possible mechanism of morphology-change after Joule-annealing and doping.

Figure S5: Raman spectrum of F4TCNQ.


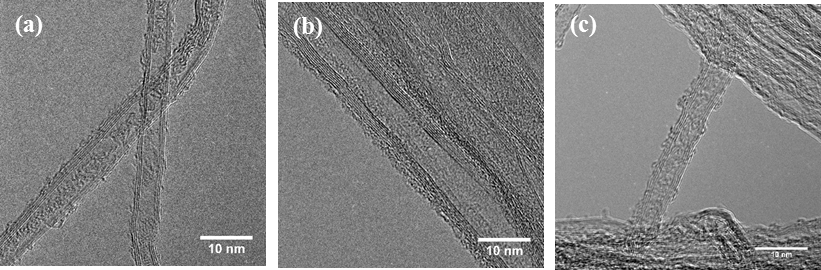


Figure S6: TEM images of (a) pristine, (b) Joule-annealed, and (c) Joule-annealing followed by 2.5 mg/ml F4TCNQ doped FWCNT yarns.

**
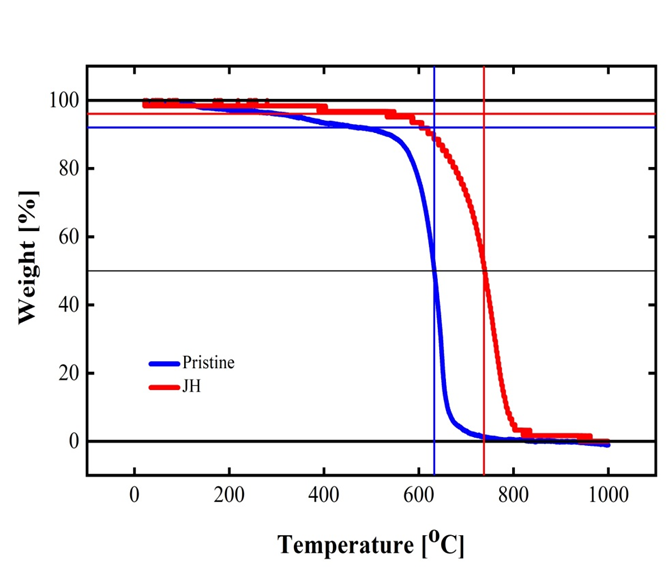
**

Figure S7: TGA analysis of the FWCNT yarn before and after Joule-annealing.
